# Supplementary material for: A Retrospective Medical Record Review of Adults with Non-Cancer Diagnoses Prescribed Medicinal Cannabis
Source: J Clin Med. 2023 Feb 13;12(4):1483. doi: 10.3390/jcm12041483 (PMC9965412; doi:10.3390/jcm12041483)
Supplement: Supplementary file 1 [file jcm-12-01483-s001.zip › jcm-2113675-supplementary.pdf]

Journal of
Clinical Medicine

MDPI

Supplementary Material

Table S1. All patient conditions.

Condition(s) treated	Number of patients (%) [a] (*n* = 157)
**Musculoskeletal conditions**	39 (24.8)
Spondylosis	25 (15.9)
Osteoarthritis	6 (3.8)
Osteoporosis	2 (1.3)
Vertebral Fractures	2 (1.3)
Osteomyelitis	1 (0.6)
Mixed arthritic	1 (0.6)
Myositis	1 (0.6)
Nociceptive spine pain	1 (0.6)
**Neurological conditions**	91 (58.0)
Parkinson's disease	23 (14.6)
Migraine	16 (10.2)
Multiple sclerosis	10 (6.4)
Peripheral neuropathy	9 (5.7)
Neuropathic pain	9 (5.7)
Epilepsy	3 (1.9)
Complex regional pain syndrome	2 (1.3)
Axonal neuropathy	2 (1.3)
Stiff person syndrome	2 (1.3)
Dystonia	2 (1.3)
Post herpetic neuralgia	1 (0.6)
Trigeminal neuralgia	1 (0.6)
Posterior femoral cutaneous nerve lesion	1 (0.6)
Lower limb spastic paresis	1 (0.6)
Thoracic paraplegia	1 (0.6)
Tardive dyskinesis	1 (0.6)
Ischaemic stroke	1 (0.6)
Corticobasal degeneration	1 (0.6)
Myelitis	1 (0.6)
Myelopathy	1 (0.6)
Multiple systems atrophy	1 (0.6)
Phantom limb syndrome	1 (0.6)
Facial onset sensory and motor neuronopathy	1 (0.6)
**Autoimmune conditions**	14 (8.9)
Systemic lupus erythematosus	3 (1.9)
Fibromyalgia[b]	3 (1.9)
Sjogren's syndrome	2 (1.3)
Polymyalgia rheumatica[c]	2 (1.3)
Immune arthritis	2 (1.3)
Scleroderma	1 (0.6)
Rheumatoid arthritis	1 (0.6)
**Inflammatory conditions**	10 (6.4)
Endometriosis	3 (1.9)
Arachnoiditis	2 (1.3)

Rectal abscess	1 (0.6)
Interstitial cystitis	1 (0.6)
Ulcerative colitis	1 (0.6)
Pancreatitis	1 (0.6)
Chronic sinusitis	1 (0.6)
**Mental health conditions**	3 (1.9)
Insomnia	2 (1.3)
Post-traumatic stress disorder	1 (0.6)
**Other**	23 (14.6)
Chronic pain syndrome	19 (12.1)
Chronic abdominal pain	2 (1.3)
Renal failure	1 (0.6)
Headaches	1 (0.6)

[a] Percentages are given as individual frequencies out of the entire study population, as some patients had more than one condition percentages will add to over 100; [b]Not considered an inflammatory disease, with recent research suggesting that fibromyalgia might be an autoimmune disease; [c]Inflammatory disorder that is believed to be an autoimmune disease.

Table S2. All patient indications.

**Indication**	**Number of patients (%)** [a] **(*n* = 157)**
Pain	136 (86.6)
Muscle spasms	18 (11.5)
Sleep	10 (6.4)
Rigidity	5 (3.2)
Spasticity	5 (3.2)
Anorexia	5 (3.2)
Dystonia	5 (3.2)
Tremors	4 (2.5)
Speech	4 (2.5)
Seizures	3 (1.9)
Dyskinesias	3 (1.9)
Bradykinesia	3 (1.9)
Anxiety	2 (1.3)
Dysphagia	2 (1.3)
Mobility	2 (1.3)
Nausea	2 (1.3)
Burning mouth syndrome	1 (0.6)
Tone	1 (0.6)
Clonus	1 (0.6)
Fatigue	1 (0.6)
Weakness	1 (0.6)
Stroke symptoms	1 (0.6)
Stiffness	1 (0.6)
Dry mouth and eyes	1 (0.6)
Restless legs	1 (0.6)
Facial onset sensory and motor neuronopathy symptoms	1 (0.6)

[a] Percentages are given as individual frequencies out of the entire study population, as some patients had more than one indication percentages will add to over 100.
